# Bone morphogenetic protein receptor 2 inhibition destabilizes microtubules promoting the activation of lysosomes and cell death of lung cancer cells

**DOI:** 10.1186/s12964-021-00743-w

**Published:** 2021-09-25

**Authors:** Arindam Mondal, Rachel NeMoyer, Mehul Vora, Logan Napoli, Zoya Syed, Elaine Langenfeld, Dongxuan Jia, Youyi Peng, John Gilleran, Jacques Roberge, Christopher Rongo, Salma K. Jabbour, John Langenfeld

**Affiliations:** 1 Department of Surgery, Rutgers Robert Wood Johnson Medical School, Rutgers, The State University of New Jersey, New Brunswick, NJ 08903 USA; 2 Department of Genetics, Rutgers University, Piscataway, NJ 08854 USA; 3 Rutgers University, Piscataway, NJ 08854 USA; 4 Biomedical Informatics Shared Resources, Rutgers Cancer Institute of New Jersey, New Brunswick, NJ 08903 USA; 5 Molecular Design and Synthesis, Rutgers University, Piscataway, NJ 08854 USA; 6 Department of Radiation Oncology, Rutgers Robert Wood Johnson Medical School, New Brunswick, NJ 08903 USA

## Abstract

**Background:**

Recent studies have shown that bone morphogenetic protein receptor 2 (BMPR2) regulates cell survival signaling events in cancer cells independent of the BMP type 1 receptor (BMPR1) or the Smad-1/5 transcription factor. Mutations in BMPR2 trafficking proteins leads to overactive BMP signaling, which leads to neurological diseases caused by BMPR2 stabilization of the microtubules. It is not known whether BMPR2 regulates the microtubules in cancer cells and what effect this has on cell survival. It is also not known whether alterations in BMPR2 trafficking effects activity and response to BMPR2 inhibitors.

**Methods:**

We utilized BMPR2 siRNA and the BMP receptor inhibitors JL5 and Ym155, which decrease BMPR2 signaling and cause its mislocalization to the cytoplasm. Using the JL5 resistant MDA-MD-468 cell line and sensitive lung cancer cell lines, we examined the effects of BMPR2 inhibition on BMPR2 mislocalization to the cytoplasm, microtubule destabilization, lysosome activation and cell survival.

**Results:**

We show that the inhibition of BMPR2 destabilizes the microtubules. Destabilization of the microtubules leads to the activation of the lysosomes. Activated lysosomes further decreases BMPR2 signaling by causing it to mislocalizated to the cytoplasm and/or lysosome for degradation. Inhibition of the lysosomes with chloroquine attenuates BMPR2 trafficking to the lysosome and cell death induced by BMPR2 inhibitors. Furthermore, in MDA-MD-468 cells that are resistant to JL5 induced cell death, BMPR2 was predominately located in the cytoplasm. BMPR2 failed to localize to the cytoplasm and/or lysosome following treatment with JL5 and did not destabilize the microtubules or activate the lysosomes.

**Conclusions:**

These studies reveal that the inhibition of BMPR2 destabilizes the microtubules promoting cell death of cancer cells that involves the activation of the lysosomes. Resistance to small molecules targeting BMPR2 may occur if the BMPR2 is localized predominantly to the cytoplasm and/or fails to localize to the lysosome for degradation.

**Video Abstract**

**Supplementary Information:**

The online version contains supplementary material available at 10.1186/s12964-021-00743-w.

## Background

The bone morphogenetic protein (BMP)-signaling cascade regulates a plethora of activities throughout embryonic development, including lung development. In the mature lung there is little BMP signaling, however, BMP becomes reactivated in lung cancer and lung inflammation [1–3]. The BMP2 ligand is highly over-expressed in non-small cell lung carcinomas (NSCLC) with little expression in normal lung tissue and benign lung tumors [4]. Several studies have shown that the BMP signaling cascade has significant tumorigenesis in many different tumor types promoting cell migration, invasion, proliferation, stemness, angiogenesis, and high ligand expression correlates with a worse prognosis [3, 5–10].

There are over 20 BMP ligands that signal through serine/threonine transmembrane bone morphogenetic protein receptor (BMPR) type 1 and type 2. Ligands bind BMP type 1 receptors (alk2, alk3, or alk6), which are then phosphorylated and activated by a constitutively active BMP type 2 receptor (BMPR2, ActR-IIA, ActRIB) [11]. BMP ligands are subclassed based on sequence similarities that differ in their affinity for the BMP receptors and activation of downstream signaling events [12]. The BMPR1/BMPR2 complex phosphorylates the Smad-1/5 transcription factor, activating downstream targets including the inhibitor of differentiation proteins Id1, Id2, and Id3 [13–15]. BMPR2 can also signal independent of the type 1 BMP receptors and Smad-1/5 signaling. Recent studies have shown that BMPR2 Smad-independent signaling regulates cell survival mechanisms in cancer cells. BMP inhibitors of the type 1 receptors (DMH1 (dorsomorphin homolog 1), LDN) cause little cell death of cancer cells in comparison to BMP inhibitors targeting both the type 1 and type 2 receptors (JL5, DMH2) [7, 8]. Suppression of BMPR2 with siRNA knockdown or JL5 causes an increase release of the mitochondrial proteins Smac/DIABLO and cytochrome c into the cytosol [16]. BMPR2 Smad-independent signaling regulates potent anti-apoptotic proteins including transforming growth factor beta (TGFβ), transforming growth factor beta-activated kinase 1 (TAK1), and anti-apoptotic proteins X-linked inhibitor of apoptosis protein (XIAP) [7, 16]. BMPR2 Smad-independent signaling also regulates actin polymerization by activating LIM kinase [17] and chemotaxis through the activation of PI3 kinase [18]. The mechanisms by which the inhibition of BMPR2 initiates cell death are not known.

Mutations that prevent trafficking of BMPR2 to the lysosomes for degradation lead to over activity of BMPR2 signaling, leading to paralysis and neurodegeneration [19–21]. The mechanism by which enhanced BMPR2 activity leads to these neurological events is caused by BMPR2 stabilizing the microtubules [19, 20]. BMPR2 has a long cytoplasmic tail that binds and regulates cytoskeletal proteins actin and the microtubules [17]. BMPR2 stabilization of cytoskeletal proteins mediates a number of developmental processes, including cell migration [22–24]. Despite studies linking neurological diseases to alterations in BMPR2 trafficking, mediated by the stabilization of the microtubules, the role of BMPR2 regulation of microtubules in cancer cells and its effect on survival has never been studied. Furthermore, it is not known whether different cancer types have differences in trafficking of BMPR2 affecting its activity and/or response to BMPR2 inhibitors.

The present study examines whether the inhibition of BMPR2 destabilizes the microtubules in cancer cells and whether this effects survival. In addition, we also examined whether sensitivity to the BMP inhibitor JL5 was dependent on its ability to cause mislocalization of BMPR2 to the cytosol and/or lysosome for degradation. These studies reveal that inhibition of BMPR2 causes the destabilization of the microtubules in lung cancer cells, which affects survival by activating the lysosomes. MDA-MB-468 cells were found to be resistant to JL5 induced cell death, destabilization of the microtubules, and activation of the lysosomes. MDA-MB-468 cells have few cells that expressed BMPR2 at the plasma membrane and JL5 did not cause mislocalization of BMPR2 to the cytoplasm or lysosome, which occurs in sensitive lung cancer cells. These studies reveal that inhibition of BMPR2 mediates cell death in part by its regulation of the microtubules. These studies also suggest that resistance to BMPR2 inhibitors may occur in cancer cells that do not express BMPR2 at the plasma membrane and/or the inhibitor does not cause BMPR2 to be mislocalized to the cytoplasm and/or lysosome for degradation.

## Methods

### Cell culture and reagents

The immortalized A549 and H1299 cell lines obtained from patients with non-small cell lung cancer (ATCC) were cultured in Dulbecco’s modified Eagle’s medium (DMEM, Sigma Aldrich, Saint Louis, MO, USA) with 5% fetal bovine serum. The immortalized MDA-MB-468 cell line (ATCC) obtained from a patient with breast cancer was cultured in RPMI media supplemented with 10% FBS. JL5 was synthesized by John Gilleran and Jacques Roberge from Rutgers Molecular Design and Synthesis. Ym155 and Z-VAD-FMK were purchased from Selleckchem (Houston, TX USA) and R&D Systems (Minneapolis, MN USA), respectively. Chloroquine was purchased from Sigma Aldrich (Saint Louis, MO, USA). List of antibodies with catalog numbers and dilutions and assay kits can be found in Additional file 1: Table S1.

### Cell viability

450,000 cells per well were plated into 6-well plates and grown overnight. Next day, cells were treated for the designated period. An automated cell counter Vi-CELL cell analyzer (Beckman Coulter) was used to determine the cell viability. The Vi-CELL cell analyzer uses trypan blue dye in approximately 500 cells per sample to determine the number of dead cells.

### Immunofluorescence staining

400,000 cells per well for H1299 and 450,000 cells per well for A549 were seeded onto a microscope cover glass in a 6-well plate for 24 h. The cells were treated for the designated period and then washed with PBS. 4% formaldehyde for 10 min was used to fix the cells and 0.5% triton-X was used to permeabilize the cells. To analyze BMPR2 on the plasma membrane, the permeabilization step was not performed. The cells were blocked with CAS-Block (Life technologies, USA) for 1 h. Then cells were stained with the primary antibodies for 1 h according to the experiment. The primary antibodies were, rabbit polyclonal anti-BMPR2 antibody, which recognizes an extracellular epitope (Sigma-Aldrich), Alexa Fluor 594 anti-tubulin-α antibody (Biolegend, USA), AlexaFluor 488 conjugated LAMP1 (Santa Cruz Biotechnology, USA), acetylated alpha tubulin (Santa Cruz Biotechnology, USA), or rabbit monoclonal anti-Cathepsin B. After 1 h, cells were washed with PBS. For BMPR2 and acetylated alpha tubulin staining, cells were stained with Alexa Flour 488 conjugated secondary antibody for 1 h at room temperature. Then the cells were washed with PBS and counterstained with DAPI (Sigma-Aldrich) for 10 min. Cells were washed with PBS and distilled water. Cover glasses were mounted upside down with a mounting media on a microscope slide and dried overnight at room temperature in the dark. The stained cells were observed using 60X oil lens using a fluorescence microscope (Nikon eclipse TE300).

### Co-localization analysis

For the analysis of BMPR2 and LAMP1 co-localization, ImageJ software (NIH, USA) with JaCOP extension was used. A manual threshold was chosen and according to that manual threshold, red and green fluorescence was selected. Mander’s co-efficient (M1) values were also obtained from the ImageJ software where the fraction of A (BMPR2-red) overlapping B (LAMP1-Green) were calculated. The mean values with the standard error of the mean (SEM) were plotted as a histogram.

### Green Cathepsin B staining

Green Cathepsin B assay kit was purchased from ImmunoChemistry Technologies (Bloomington, MN, USA). The staining was performed according to manufacturer’s protocol. Briefly, 400,000 cells per well for H1299 and 450,000 cells per well for A549 were seeded onto a microscope cover glass in a 6-well plate for 24 h. The cells were then treated for a designated period and then washed with PBS. The media was changed and the diluted R110-(RR)2 was added into the media. The cells were incubated at 37 °C in the dark for 1 h and then washed with PBS once. 4% formaldehyde was used for 10 min to fix the stained cells. After fixing, the cells were washed with PBS and then with distilled water. Cover glasses were mounted upside down and dried overnight. The stained cells were observed using 60X oil lens under a fluorescence microscope (Nikon eclipse TE300).

### LysoTracker™ green staining

LysoTracker™ Green DND-26 was purchased from Thermo Fisher Scientific, USA and the staining was performed according to the manufacturer’s protocol. Briefly, 400,000 cells per well for H1299 and 450,000 cells for A549 were seeded onto a microscope cover glass in a 6-well plate for 24 h. The cells were then treated for the designated time and then washed with PBS. 75 nM dye in 1 ml media was added to each well and incubated for 5 min at 37 °C. 4% formaldehyde was used for 10 min to fix the stained cells. After fixing, the cells were washed with PBS and distilled water. Cover glasses were mounted upside down and dried overnight. The stained cells were observed using 60X oil lens under a fluorescence microscope (Nikon eclipse TE300).

### Transient knockdown

Validated select siRNAs (Life Technologies, ID: s2044 and s2045) were used for BMPR2 knockdown studies. To evaluate the selectivity, Silencer Select negative control siRNA (4,390,843) was used. Lipofectamine® RNAiMAX Reagent (Invitrogen, Carlsbad, CA, USA) was used to transfect the siRNA. 750,000 cells were plated in a 6-well plate and were grown overnight. The next morning the cells were transfected with 6 nM BMPR2 or 6 nM of siRNA control according to manufacturer’s protocol.

### Western blot analysis

Cells were treated with the designated period and then cellular protein was extracted using RIPA buffer. Protein concentration was determined using BCA method. Equal amount of protein was loaded into polyacrylamide gel and separated by SDS-PAGE (sodium dodecyl sulfate polyacrylamide gel electrophoresis). The proteins were transferred to nitrocellulose membrane (Schleicher and Schuell, Keene, NH). HRP (horseradish peroxidase) conjugated primary antibodies were used overnight at 4 °C. Primary antibodies used in this study were rabbit monoclonal anti-XIAP, rabbit monoclonal anti-PARP, rabbit monoclonal anti-pSmad1/5, (Cell signaling Technology, MA, USA), rabbit monoclonal anti-Id1 (Calbioreagents, San Mateo, CA), rabbit anti-actin, an affinity isolated antigen specific antibody (Sigma, Saint Louis, MO), and rabbit polyclonal anti-GAPDH (Sigma, Saint Louis, MO).

### Statistical analysis

Paired student t-test assuming unequal variances was used for statistical analysis. The mean of control was compared with the mean of each treated group. Differences with p values < 0.05 were considered statistically significant.

## Results

### Ym155 decreases BMP signaling in lung cancer cells

**Fig. 1:**
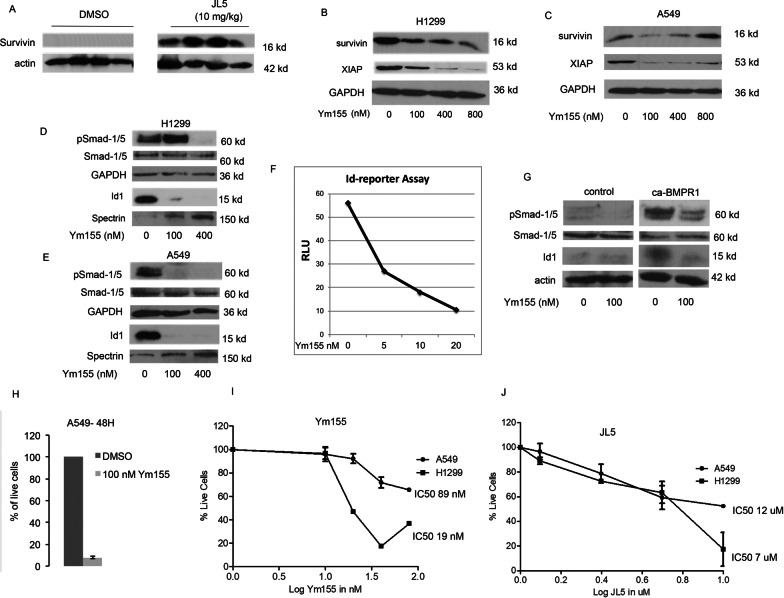
Ym155 regulates BMP signaling in lung cancer cells. **A** Immunoblot of tumors treated with JL5 for 4 days. **B**, **C** Western blot analysis of cells treated with Ym155 for 24 h. **D**, **E** Western blot analysis of BMP signaling following treatment with Ym155 for 24 h. **F** H1299 cells stably transfected with Id1-luciferase reporter were treated with Ym155 for 24 h and relative luminescence units (RVU) measured. Data shown is the mean of 2 independent experiments. **G** H1299 cells were transiently transfected with control vector or caBMPR1 then treated with Ym155. Western blot analysis of transfected cells treated with Ym155 for 24 h. **H** Data shows the mean of percent live cells of 3 independent experiments after being treated with Ym155 for 48 h. **J** Dose response curves of the number of live cells after being treated with Ym155 (**I**) or JL5 for 24 h

We previously reported that JL5 decreases the expression of Id1 but not XIAP in tumor xenografts [8]. Probing a blot from this prior study showed an increased expression of Survivin in tumors treated with JL5 for 5 days (Fig. 1A). Survivin belongs to the family of anti-apoptotic proteins that have been reported to be regulated by BMP signaling and are known to stabilize the expression of XIAP [25].

We explored the effects of Ym155, a reported survivin inhibitor [26], on the expression of survivin and XIAP in lung cancer cells. Ym155 induced a significant decrease in the expression of XIAP in H1299 cells (Fig. 1B) and A549 cells (Fig. 1C) with little change in the expression of survivin. This is consistent with prior studies suggesting that Ym155 mediates its effects independent of survivin [27].

Since BMPR2 regulates XIAP through Smad-1/5 independent mechanisms, we examined whether Ym155 regulated BMP signaling. The effects of Ym155 on BMP Smad-1/5 dependent signaling was assessed by examining changes in the phosphorylation of the BMP transcription factor Smad-1/5 and its downstream transcriptional target Id1. Ym155 caused a significant decrease in the activity of Smad-1/5 and Id1 in both the H1299 (Fig. 1D) and A549 (Fig. 1E) cells at nanomolar concentrations. H1299 cells were stably transfected with the Id1 promoter driving the expression of the luciferase reporter. Ym155 caused a dose responsive decrease in the Id1 reporter (Fig. 1F). Transfection of constitutively active BMPR1A (caBMPR1A) activates Smad-1/5-Id1 signaling. Ym155 inhibited caBMPR1A activation of Smad-1/5 (Fig. 1G). These studies suggest that Ym155 inhibits both Smad-1/5 dependent and independent signaling.

We examined the effects of Ym155 on cell survival and the induction of cell death. After 48 h the majority of A549 cells treated with 100 nM were dead (Fig. 1H). Examining the IC50 at 24 h with concentrations below 100 nM, we found the IC50 to be 89 nM and 19 nM for A549 and H1299, respectively (Fig. 1I). In comparison, the BMP inhibitor JL5’s IC50 was higher with an IC50 of 12 μM and 7 μM for A549 and H1299 cells, respectively (Fig. 1J).

### MDA-MB-468 cells are resistant to JL5

**Fig. 2:**
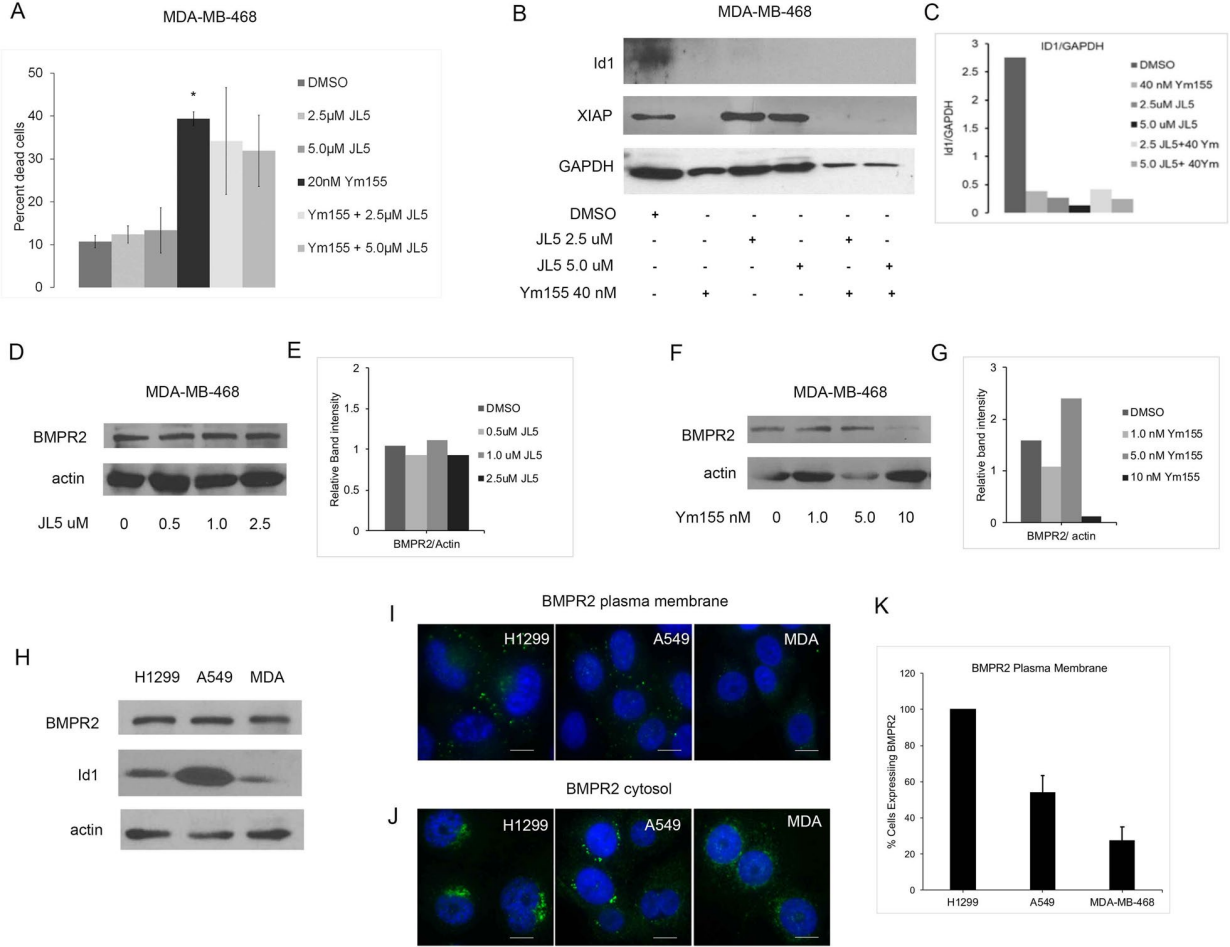
MDA-MB-468 cells are resistant to JL5 and sensitive to Ym155. **A** Cell counts of MDA-MB-468 cells treated for 24 h with JL5 and Ym155 alone and in combination**.** **B**–**G** Western blot analysis of Id1, XIAP, BMPR2 in MDA-MB-468 cells treated for 24 h and densitometric analysis of expression relative to loading control. **H** Immunoblot for BMPR2 of untreated cell lines. **I**–**J** Representative immunofluorescent images of BMPR2 at the plasma membrane and in the cytosol in the different cell lines. **K** Quantification of the number of cells that expressed BMPR2 at the plasma membrane. Approximately 50 cells from each cell line were examined for BMPR2 expression on the plasma membrane. Data is reported as the mean number of cells expressing BMPR2 at the plasma membrane. Each scale bar represents 10 μM

Breast cancer cells lacking estrogen, progesterone, and human epidermal growth factor receptor 2 (HER2) (triple negative breast cancer) are highly aggressive tumors that are resistant to cancer therapeutics. We identified that the triple negative breast cancer cell line (MDA-MB-468) is resistant to growth suppression and/or cell death by the BMP inhibitor JL5 (Fig. 2A). Ym155 induced cell death of MDA-MB-468 cells and the combination with JL5 had no additional effect (Fig. 2A).

We previously reported that knockdown of BMPR2 and JL5 decreases the expression of Id1 and XIAP in H1299 and A549 cells [16]. JL5 and Ym155 decreased the expression of Id1 in MDA-MB-468 cells indicating suppression of Smad-1/5 dependent signaling (Fig. 2B, C). JL5 did not decrease the expression of XIAP in MDA-MB-468 cells but was significantly decreased by Ym155 (Fig. 2B, C). Ym155 but not JL5 decreased the expression of BMPR2 in MDA-MB-468 cells (Fig. 2D–G). These studies show that Ym155 but not JL5 suppresses BMPR2 Smad-independent signaling in MDA-MB-468 cells.

### BMPR2 is expressed in the cytosol in MDA-MB-468 cells

We examined if resistance to JL5 in MDA-MB-468 cells occurred at the level of BMPR2. By Western blot analysis, there was equivalent expression of BMPR2 between the lung cancer cell lines and MDA-MB-468 cells (Fig. 2H). JL5 induces cell death in both the H1299 and A549 cells but the H1299 cells are more responsive [8]. Using an antibody that recognizes an external epitope of BMPR2, we examined the expression of BMPR2 at the plasma membrane. To assess BMPR2 in the cytosol, the cells were permeabilized prior to immunostaining. All of the H1299 cells expressed BMPR2 at the cell surface (Fig. 2I–K). Approximately 60% of A549 cells expressed BMPR2 at the cell surface but all of the cells expressed it in the cytosol (Fig. 2I–K). Only approximately 30% of MDA-MB-468 cells expressed BMPR2 at the cell surface and all the cells expressed it in the cytosol (Fig. 2I–K). These studies suggest that the level of expression of BMPR2 at the plasma membrane determines whether cancer cells are resistant or sensitive to JL5.

### JL5 decreases expression of BMPR2 on the plasma membrane in sensitive lung cancer cells but not MDA-MB-468 cells

**Fig. 3:**
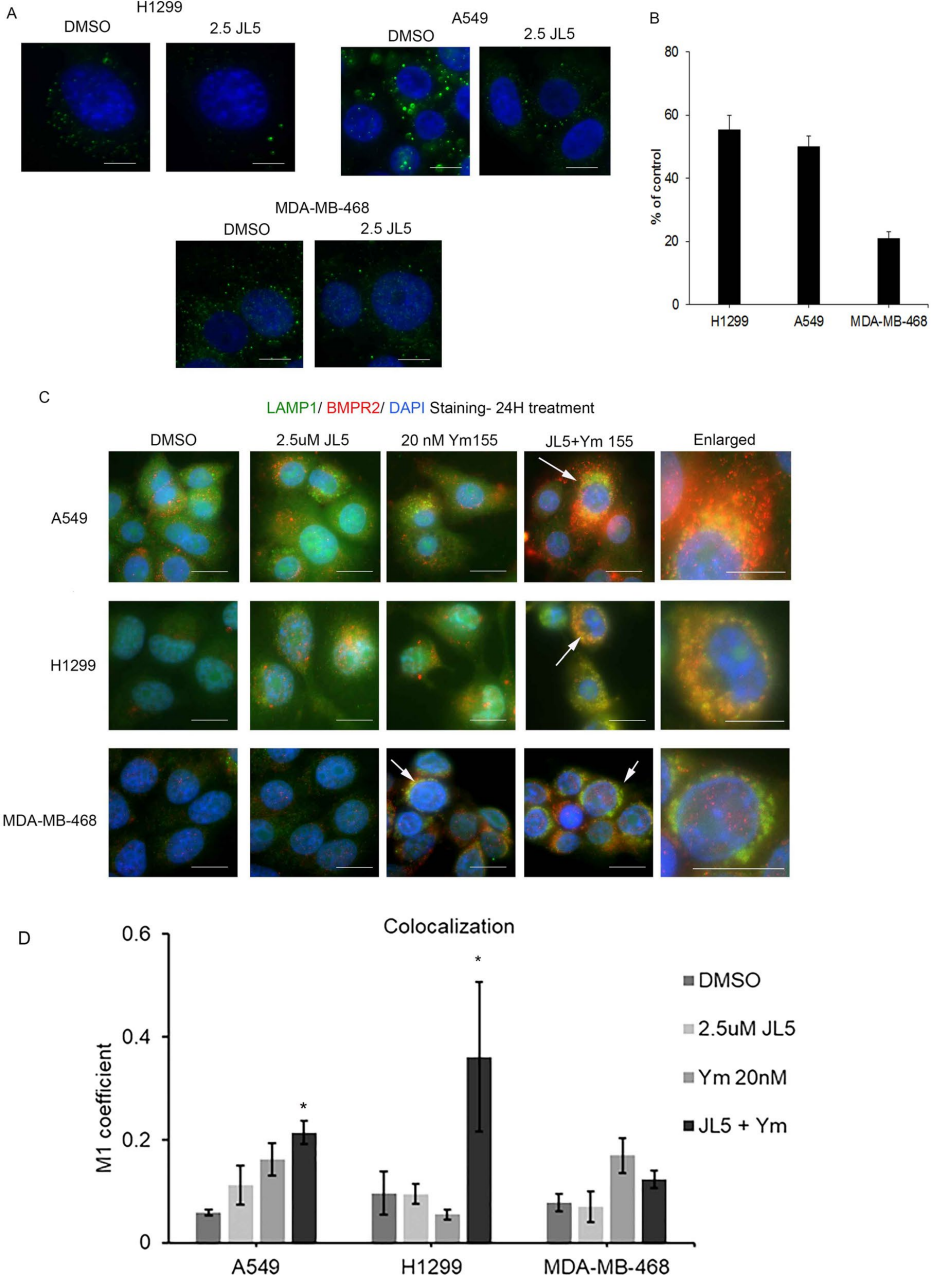
JL5 induces trafficking of BMPR2 from the plasma membrane in sensitive lung cancer cell lines but not in resistant MDA-MB-468 cells. **A** Immunofluorescence imaging of BMPR2 on the plasma membrane treated with vehicle control or JL5 for 24 h. **B** Quantification of fluorescent intensity of BMPR2 on the plasma membrane after treatment with JL5 reported as the percent decrease in comparison to vehicle control. **C** Dual immunofluorescent staining for LAMP1 and BMPR2 24 h following treatment with JL5 and Ym155 alone and in combination. Arrows demonstrate co-localization of BMPR2 with LAMP1. **D** Co-localization of LAMP1 was quantified using Image J. *p < 0.05. Each scale bar represents 10 μM

JL5 induces cytosolic mislocalization of BMPR2 in H1299 and A549 cells [16]**.** To assess whether a response to BMP inhibitors is dependent on BMPR2 being mislocalized from the plasma membrane, the number of cells expressing BMPR2 at the plasma membrane before and after treatment with JL5 for 24 h was determined. Since not all untreated cells express BMPR2 at the plasma membrane, we recorded results as percent change from control. There was approximately a 50% decrease in the expression of BMPR2 on the plasma membrane following treatment with JL5 in both H1299 and A549 cells (Fig. 3A, B). In the MDA-MB-468 cells, JL5 caused little change in the expression of BMPR2 on the plasma membrane. These studies suggest that sensitivity to JL5 depends on mislocalization of BMPR2 from the plasma membrane.

### JL5 together with Ym155 causes localization of BMPR2 to the lysosomes in lung cancer cells but not MDA-MB-468 cells

We examined whether Ym155 and JL5 alone or in combination induced BMPR2 localized to the lysosome. Immunostaining demonstrates that BMPR2 co-localizes with the lysosomal marker lysosomal-associated membrane protein 1 (LAMP1) in H1299 and A549 cells treated with JL5 and Ym155 in combination but not with each compound alone (Fig. 3C, D). In the MDA-MB-468 cells, BMPR2 co-localized with LAMP1 only when treated with Ym155, which did not enhance further with JL5 (Fig. 3C, D). These studies suggest that JL5 and Ym155 when used in combination enhance the downregulation of BMP signaling by causing BMPR2 to localize to the lysosome for degradation.

### Inhibition of BMPR2 activates lysosomes

**Fig. 4:**
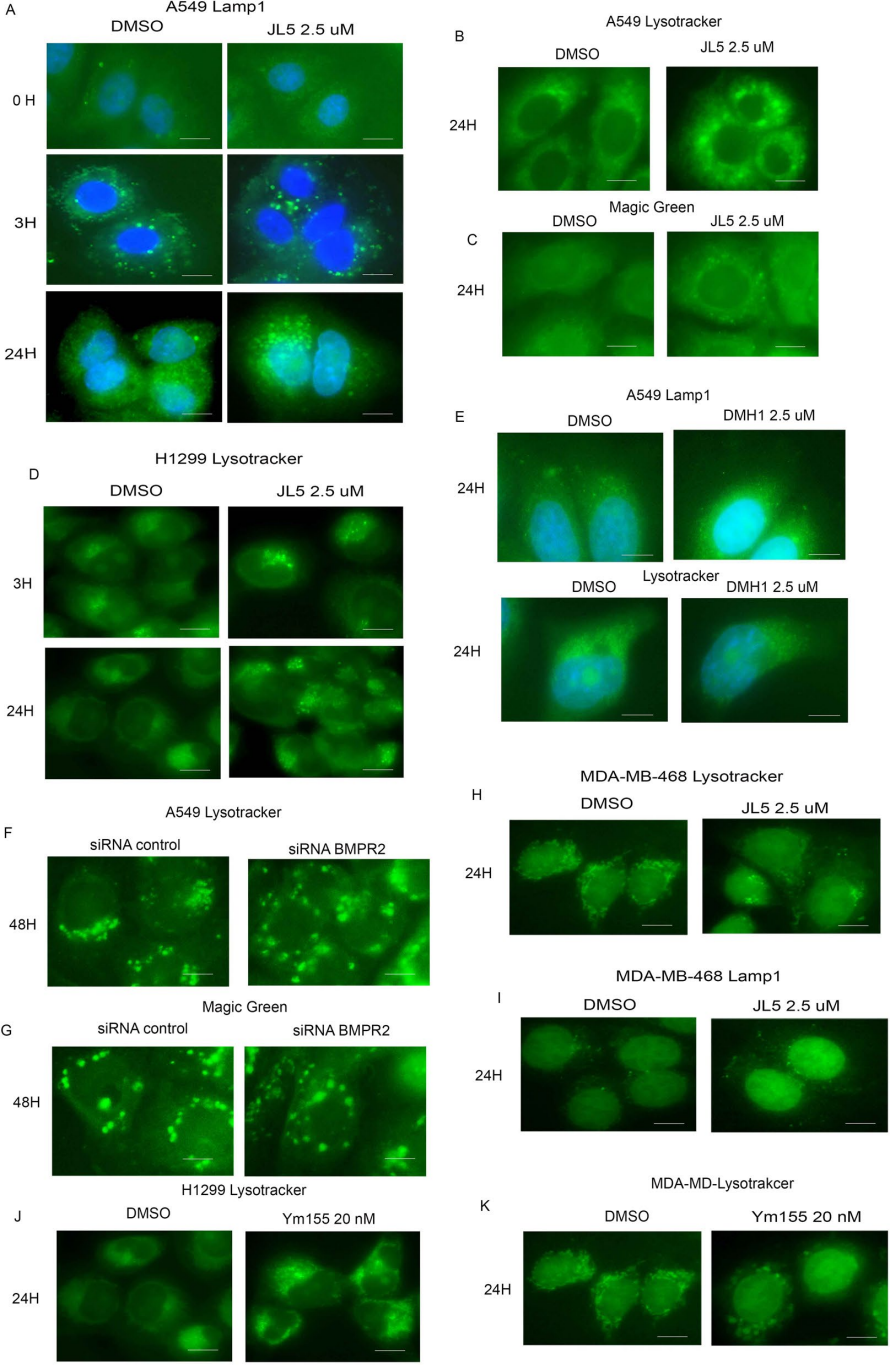
Inhibition of BMPR2 activates lysosomes in sensitive lung cancer cell lines but not in MDA-MB-468 cells. **A** Immunofluorescence imaging of LAMP1 in treated A549 cells treated with JL5. (B-C) Immunofluorescence imaging for Lysotracker™ Green and Green Cathepsin B of A549 treated with JL5 for 24 h. **D** Immunofluorescence imaging of Lysotracker™ Green of H1299 treated with JL5. **E** Immunofluorescence imaging for LAMP1 and Lysotracker™ Green of cells treated with DMH1 for 24 h. **F**–**G** Immunofluorescence imaging for Lysotracker™ Green and Green Cathepsin B of A549 cells transfected with control or BMPR2 siRNA for 48 h**.** **H**, **I** Immunofluorescence imaging for Lysotracker™ Green and LAMP1 of MDA-MB-468 cells treated for 24 h with JL5. (J-K) Immunofluorescence imaging Lysotracker™ Green of H1299 and MDA-MD-468 cells treated with Ym155 for 24 h. Each scale bar represents 10 μM

We asked whether the enhanced localization of BMPR2 to the lysosome induced by JL5 and Ym155 was caused by the inhibition of BMPR2 affecting lysosome activity. Lysosomes are normally located in the perinuclear region. Immunostaining with LAMP1 demonstrated that within 3 h, JL5 causes the lysosomes to move away from the perinuclear region and into the cytoplasm (Fig. 4A). By 24 h, the lysosomes were significantly larger in cells treated with JL5 (Fig. 4A). Lysotracker™ is fluorescent in an acidic environment and is used as a marker of the acidification of late endosomes and lysosomes. Lysotracker™ also demonstrated that JL5 caused the lysosome to become larger and localize throughout the cytoplasm in A549 (Fig. 4B) and H1299 cells (Fig. 4D). Lysotracker™ intensity was also greater following treatment with JL5 demonstrating increased acidification. Green Cathepsin B assay was used to measure activity of the lysosome protease Cathepsin B. An increase in Cathepsin B activity was seen after treatment with JL5 (Fig. 4C).

The BMP inhibitor DMH1 targets only the BMP type 1 receptors and does not cause cytosolic localization of BMPR2 [16]**.** DMH1 did not affect the localization or size of the lysosome as determined by LAMP1 staining and Lysotracker™ (Fig. 4E). Knockdown of BMPR2 in A549 cells caused an increased in the size and cytoplasmic localization of the lysosomes. BMPR2 knockdown also increased fluorescence of Lysotracker™ and increased Cathepsin B activity (Fig. 4F–G). In the MDA-MB-468 cells, JL5 had no effect on the lysosomes localization, size, or acidification (Fig. 4H, I). However, Ym155 caused an increase in size and cytosolic localization of the lysosome in H1299 (Fig. 4J) and MDA-MB-468 cells (Fig. 4K). Ym155 also increased the fluorescence of Lysotracker™ suggesting an increase in acidification. These studies suggest that inhibition of BMPR2 leads to the acidification and activation of lysosomes.

### Inhibition of BMPR2 destabilizes the microtubules

**Fig. 5:**
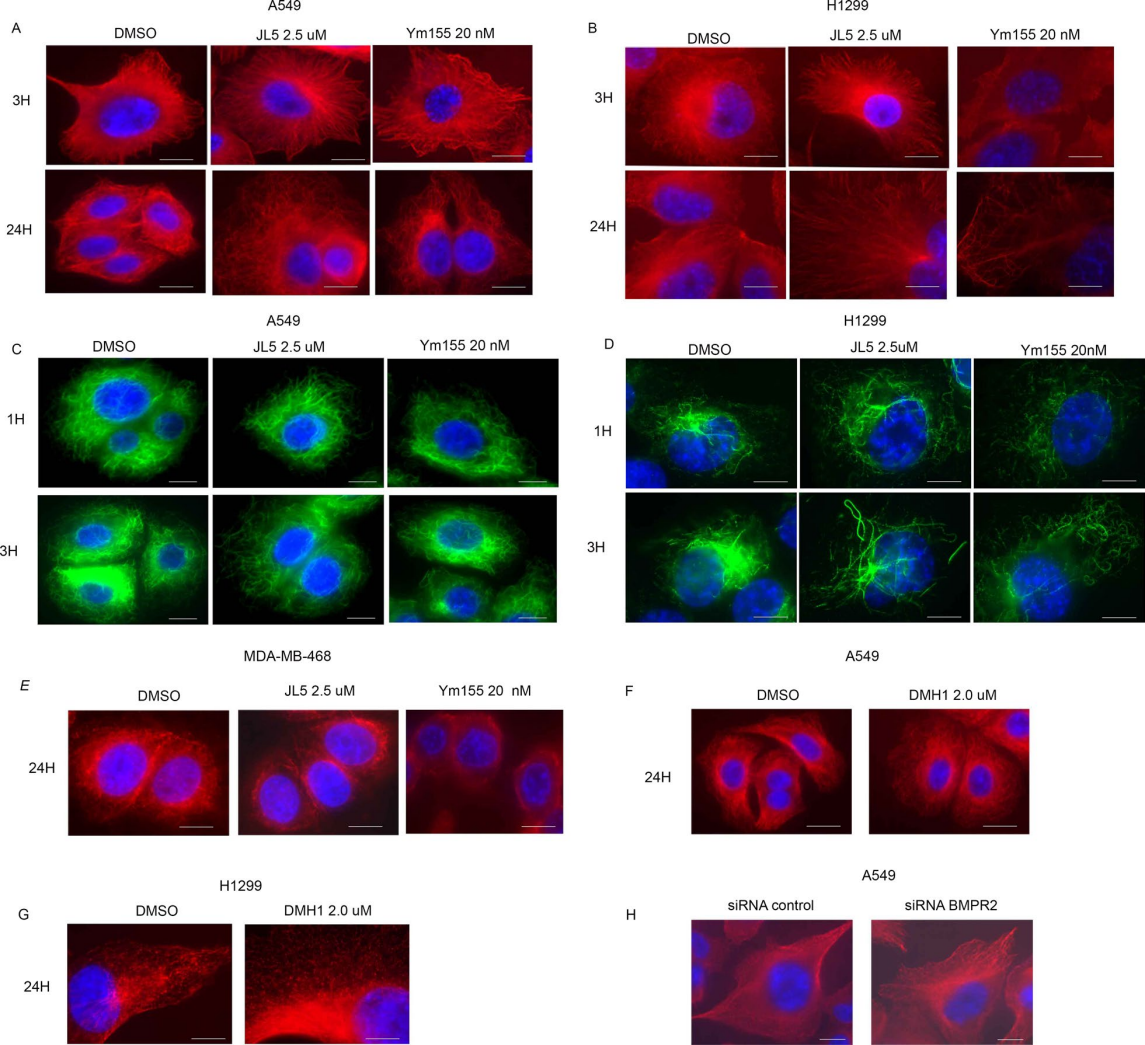
Inhibition of BMPR2 destabilizes the microtubules. **A**, **B** Immunofluorescence imaging for α-tubulin of A549 and H1299 cells treated with JL5 or Ym155. **C**, **D** Immunofluorescence imaging for acetylated α-tubulin of A549 and H1299 cells treated with JL5 or Ym155. **E** Immunofluorescence imaging for α-tubulin in MDA-MB-468 cells treated with JL5 or Ym155. **F**, **G** Immunofluorescence imaging for α-tubulin in A549 and H1299 cells treated with the BMPR1 inhibitor DMH1. **H** Immunofluorescence imaging for α-tubulin following knockdown of BMPR2 after 48 h. Each scale bar represents 10 μM

Since microtubules regulate the localization and activity of the lysosomes, we examined the effects of JL5 and Ym155 on the microtubules. Immunofluorescent imaging of α-tubulin demonstrates that JL5 and Ym155 cause significant thinning and change in the orientation of α-tubulin in A549 and H1299 cells within 3 h and progresses over time. (Fig. 5A, B). Acetylation of the microtubules is increased in polymerized microtubules [28]. Since acetylation occurs on α-tubulin, which is altered during depolymerization, we used acetylated α-tubulin staining to assess changes in microtubules. Both JL5 and Ym155 caused thinning and significant change in the orientation of acetylated α-tubulin (Fig. 5C, D). The changes in α-tubulin are consistent with JL5 and Ym155 destabilizing the microtubules in JL5 sensitive lung cancer cell lines.

JL5 did not alter the stabilization of the microtubules in MDA-MB-468 cells (Fig. 5E). Ym155 did cause significant thinning of α-tubulin in MDA-MB-468 cells (Fig. 5E). To examine the role of BMPR2 in the regulation of microtubules, lung cancer cells were treated with DMH1. DMH1 did not cause the destabilization of the microtubules in A549 or H1299 cells (Fig. 5F, G). Knockdown of BMPR2 with siRNA destabilized the microtubules (Fig. 5H). We were unable to decrease expression of BMPR2 in MDA-MB-468 cells with siRNA, so BMPR2 knockdown studies were not performed in these cells.

### Destabilizing microtubules activates lysosomes

**Fig. 6:**
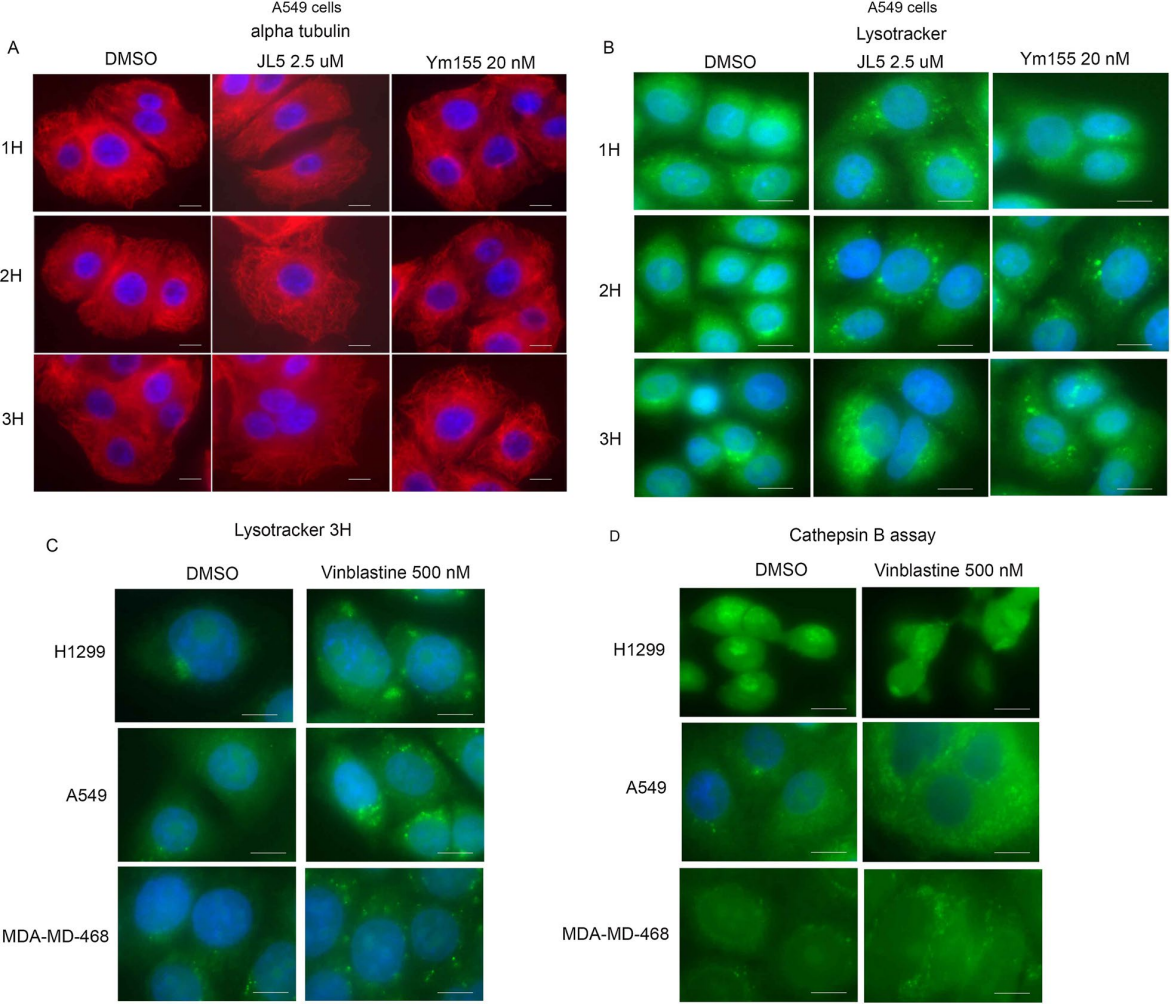
Destabilization of the microtubules activates lysosomes. **A**, **B** Immunofluorescence imaging for α-tubulin and Lysotracker™ Green of A549 cells treated with JL5 and Ym155 for 1–3 h. **C**, **D** Immunofluorescence imaging for Lysotracker™ Green and Green Cathepsin B of cells treated with Vinblastine for 3 h. Each scale bar represents 10 μM

We next asked whether destabilization of the microtubules precedes the acidification and activation of lysosomes following treatment with either JL5 or Ym155. Destabilization of the microtubules was clearly seen within 2 h following the treatment with either JL5 or Ym155 (Fig. 6A). There was a small increase in the acidification of the lysosome at 2 h, which significantly increased after 3 h (Fig. 6B). Since the changes in the microtubules were significantly more prominent at 2 h compared to the change in lysosome acidification, this suggested that microtubule depolymerization precedes the increase in lysosome acidification. To test whether destabilizing the microtubules increased the acidification and Cathepsin B proteolytic activity of the lysosomes, cells were treated with the microtubule destabilizing chemotherapeutic agent Vinblastine. Vinblastine caused a significant increase in the acidification and proteolytic activity of the lysosomes in the H1299, A549, and MDA-MB-468 cells (Fig. 6C, D). These studies suggest that the destabilization of the microtubules induced by the inhibition of BMPR2 activates the lysosomes.

### Regulation of BMPR2 by the lysosomes

**Fig. 7:**
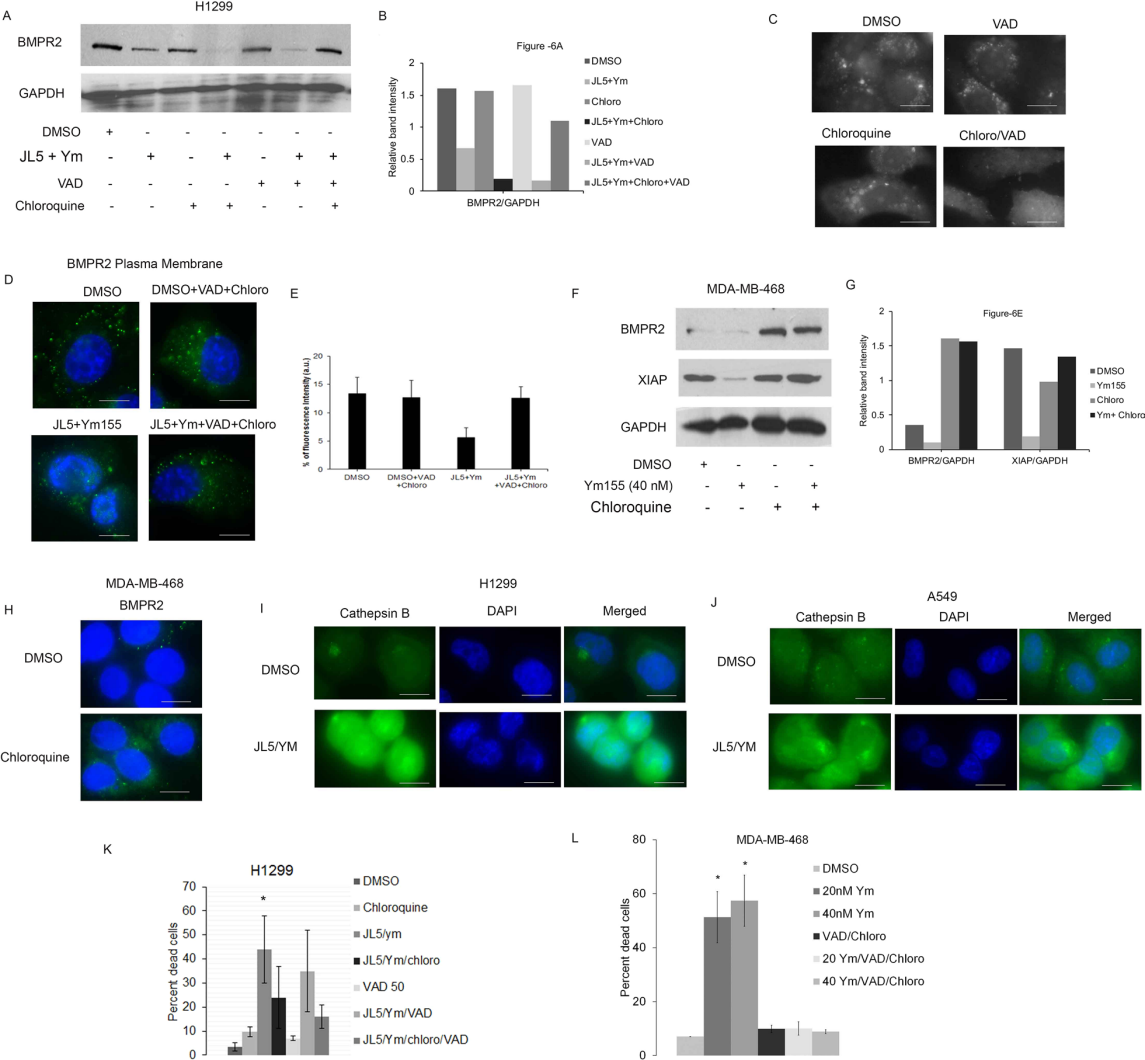
JL5 and Ym155 activation of the lysosomes regulates BMPR2 degradation and lysosomal mediated cell death. **A** Western blot analysis for BMPR2 with JL5 combined with Ym155 with and without VAD and chloroquine. **B** Densitometric analysis of BMPR2 relative to GAPDH expression. **C** Immunofluorescence imaging for Green Cathepsin B of A549 cells treated with chloroquine (chloro) and VAD alone and in combination. **D**, **E** Immunofluorescence imaging for BMPR2 at the plasma membrane of H1299 cells treated for 24 h with JL5 in combination with Ym155 with and without VAD + chloroquine. **F** Western blot of MDA-MB-468 cells treated with Ym155 with and without chloroquine and **G** densitometric analysis of expression relative to loading control. **H** Immunonofluorescence imaging of BMPR2 at the plasma membrane treated with chloroquine for 24 h. **I**, **J** Immunofluorescence imaging for Green Cathepsin B of H1299 and A549 cells treated with JL5 + Ym155 for 24 h. **K**, **L** Cell counts demonstrating percent dead cells of cells treated for 24 h with and without lysosome inhibitors. Cell count data represents the mean of 4 independent experiments. *represents *p* < .05 compared to vehicle controls. Each scale bar represents 10 μM

We examined whether the degradation of BMPR2 induced by JL5 and Ym155 was mediated by the lysosomes. H1299 cells were treated with JL5 and Ym155 in combination with and without the lysosome inhibitor chloroquine and/or the pan-caspase inhibitor Z-VAD-FMK (VAD). Chloroquine or VAD alone did not prevent the degradation of BMPR2 (Fig. 7A, B). However, the combination of chloroquine and VAD completely inhibited the degradation of BMPR2 caused by JL5 and Ym155 in combination (Fig. 7A, B). Although VAD is commonly used as a caspase inhibitor, it is reported to inhibit cathepsins at an equal potency [29, 30]. To test whether VAD inhibited lysosome proteolytic activity, A549 cells were treated with chloroquine or VAD alone or in combination and Cathepsin B activity was then determined. The combination of chloroquine and VAD caused a greater decrease in Cathepsin B activity than either agent alone (Fig. 7C). Chloroquine together with VAD also prevented the decrease in expression of BMPR2 at the plasma following JL5 and Ym155 treatment in lung cancer cells (Fig. 7D, E). In MDA-MB-468 cells, chloroquine alone prevented Ym155 induced decrease in expression of BMPR2 and its downstream target XIAP (Fig. 7F, G). Chloroquine alone also increased the localization of BMPR2 to the plasma membrane in MDA-MB-468 cells as determined by the staining of the extracellular domain of BMPR2 (Fig. 7H). Together, these studies demonstrate that activation of the lysosomes induced by JL5 and Ym155 mediates proteolytic degradation of BMPR2.

### Inhibition of lysosomes decreases cell death induced by JL5 and Ym155

An increase in lysosome permeability with the release of enzymes into the cytosol can lead to uncontrolled proteolysis and cell death [31–33]. The release of cathepsins into the cytoplasm is indicative of an increase in lysosome permeability. H1299 and A549 treated with JL5 and Ym155 in combination demonstrated a significant increase in Cathepsin B proteolytic activity as determined by Green Cathepsin B assay (Fig. 7I, J). The combination of JL5 and Ym155 also increased expression of Cathepsin B protein in the cytoplasm in both the H1299 and A549 cells (Additional file 2: Fig. S1A-B). H1299 cells were treated with JL5 and Ym155 with and without chloroquine and/or VAD and cell death was determined. Consistent with our prior studies, VAD alone had no effect on cell death induced by JL5 and Ym155. The combination of chloroquine and VAD caused a significant decrease in cell death induced by JL5 and Ym155 (Fig. 7K). In MDA-MB-468 cells, the combination of chloroquine and VAD prevented Ym155 induced cell death (Fig. 7L). These data suggest that the lysosomes mediate some of the cell death signaling induced by JL5 and/or Ym155.

## Discussion

Trafficking of BMP receptors regulates activity of the BMP signaling cascade. There is continuous cycling of BMP receptors into early endosomes, which can be cycled back to the plasma membrane by recycling endosomes for continued signaling [34, 35]. BMP receptors can also be trafficked to late endosomes that are then transported to the lysosomes for degradation, ending signaling events [34]. In hereditary spastic paraplegia (HSP) and Huntington’s disease there are mutations in BMPR2 trafficking proteins preventing BMPR2 transport to the lysosome for degradation [19–21]. This leads to enhanced BMPR2 signaling leading to neurodegeneration. We reported that JL5 induces the mislocalization of BMPR2 to the cytosol in lung cancers cells and decrease BMPR2 signaling [16]. In Caenorhabditis elegans (*C. elegans),* JL5 also decreased BMP signaling and induced localization of BMPR2 (*daf-4*) to the cytoplasm [16]. This study suggested that the mislocalization of BMPR2 to the cytoplasm is a mechanism by which JL5 suppresses BMPR2 signaling. In the present study, we show that Ym155 significantly suppresses BMP signaling and mislocalization of BMPR2 in lung cancer cell lines and MDA-MB-468 cells. MDA-MB-468 cells were found to be resistant to the growth suppressive effects of JL5 but not Ym155. MDA-MB-468 cells demonstrated less BMPR2 expression on the plasma membrane and JL5 caused significantly less trafficking of BMPR2 from the plasma membrane to the cytoplasm in comparison to sensitive lung cancer cell lines. JL5 also caused less regulation of BMPR2 downstream targets, including the regulation of XIAP, microtubule instability, and activation of the lysosomes. These studies suggest that MDA-MB-468 cells are resistant to JL5 due to the inability to suppress BMPR2 signaling.

In MDA-MB-468 cells, Ym155 decreased the expression of BMPR2, XIAP, and the BMP transcription target Id1. This suggests that BMPR2 signaling is still active in MDA-MB-468 cells. Some receptors continue to signal following internalization into early endosomes or in multi-vesicular bodies [36]. We show in this manuscript that the combination of JL5 with Ym155 promotes the trafficking of BMPR2 to the lysosomes for degradation in H1299 and A549 cells. Since Ym155 does not bind BMPR2 demonstrates that trafficking of BMPR2 is likely induced by mechanisms downstream of BMPR2 signaling. Our studies support that resistance of MDA-MB-468 is from the inability of JL5 to suppress BMPR2 signaling. JL5 only has weak inhibition of BMPR2 (IC50 8 μM) compared to its inhibition of the BMP type 1 receptors (5 nM), which may explain its lack of suppression of BMPR2 signaling in some cancer cells.

Our studies show that the inhibition of BMPR2 leads to the activation of the lysosomes. When BMPR2 activity was inhibited with JL5, Ym155, or siRNA knockdown of BMPR2, lysosomal activity was increased in lung cancer cells as demonstrated by an increase in lysosome acidification, LAMP1 expression, and Cathepsin B activity. The lysosomes were also larger and localized throughout the cytosol. In MDA-MB-468 cells, Ym155 activated lysosomes while JL5 had no effect. Activation of lysosome proteases has a number of activities in cells, including promoting receptor trafficking and degradation of macromolecules [33, 37]. An increase in lysosomal permeability (LMP) can cause uncontrolled protease activity in the cytosol leading to cell death [33]. LMP was originally thought to be an end stage event of apoptosis; however, more recent studies suggest LMP is a regulated cell death pathway via cell induced by either increased cytosolic calcium, reactive oxygen species, cell stress or hypoxia [33]. We found that the inhibition of lysosome cathepsins with chloroquine and VAD decreases cell death, prevents trafficking of BMPR2, and prevents the downregulation of BMPR2 signaling induced by JL5 and Ym155. These findings are consistent with a prior report showing that chloroquine blocks the degradation of BMPR2 and enhances BMPR2 activity in pulmonary artery endothelial cells [38]. These studies show that activated lysosomes decrease BMPR2 signaling and suggest that JL5 and Ym155 in combination induce cell death in lung cancer cells involving an increase in LMP.

BMPR2 is known to stabilize cytoskeletal proteins actin and the microtubules [17, 19, 20]. Stabilization of the microtubules by the activation of BMPR2 promotes dendrite formation and cell migration [22, 24]. Alterations in BMPR2 signaling have been shown to affect the microtubules, which is thought to contribute to the pathology of several diseases including hereditary spastic paraplegia, Huntington’s disease, and pulmonary arterial hypertension [19–21, 39]. We show that inhibition of BMPR2 signaling destabilizes microtubules in sensitive lung cancer cells. Our studies suggest that it is the destabilization of the microtubules following inhibition of BMPR2 that leads to the activation of the lysosomes. The destabilization of the microtubules induced by BMPR2 inhibition preceded the activation of the lysosomes. The microtubule-destabilizing chemotherapeutic agent Vinblastine produced a response similar to that of BMPR2 inhibition with an increase in lysosome acidification, cytosol localization, and increase in Cathepsin B activity in lung cancer cells and MDA-MB-468 cells. Similar findings were reported with the microtubule-destabilizing agent vincristine, which also increases lysosomal volume and LMP in MCF-7 breast cancer cells [40].

## Conclusions

These studies demonstrate novel mechanisms by which the inhibition of BMPR2 regulates survival in cancer cells. Our studies show that inhibition of BMPR2 destabilizes the microtubules leading to lysosome activation, which sensitizes cancer cells to LMP and cell death. Inhibition of BMPR2 has also been shown to downregulate the expression of the pro-survival proteins XIAP and TAK1. These studies suggest that BMPR2 signaling should be targeted and evaluated as a potential chemotherapeutic for the treatment of cancer.

## Supplementary Information

**Additional file 1**. JL5 and Ym155 together increase cathepsin B expression in cytoplasm.

**Additional file 2: Fig. S1**. The combination of JL5 together with Ym155 increases expression of Cathepsin B in the cytoplasm. (A-B) Immunofluorescent imaging for Cathepsin B of A549 and H1299 cells treated with JL5 2.5 uM and Ym155 20 nM alone and in combination for 24 hr. Arrows show the expression of Cathepsin B in the cytoplasm. Each scale bar represents 10 μM.

## Data Availability

The datasets obtained and analyzed for this study will be made available from the corresponding author in a reasonable request.

## Abbreviations

BMP: Bone morphogenetic protein
BMPR2: Bone morphogenetic protein receptor 2
AIF: Apoptosis inducing factor
XIAP: X-linked inhibitor of apoptosis protein
TGFβ: Transforming growth factor beta
TAK1: Transforming growth factor-beta-activated kinase 1
NSCLC: Non-small cell lung carcinomas
VAD: Z-VAD-FMK
LDN: LDN-193189
PARP: Poly ADP ribose polymerase
