# Supplementary figures and images for: Bone morphogenetic protein receptor 2 inhibition destabilizes microtubules promoting the activation of lysosomes and cell death of lung cancer cells
Source: Cell Commun Signal. 2021 Sep 25;19:97. doi: 10.1186/s12964-021-00743-w (PMC8466694; doi:10.1186/s12964-021-00743-w)

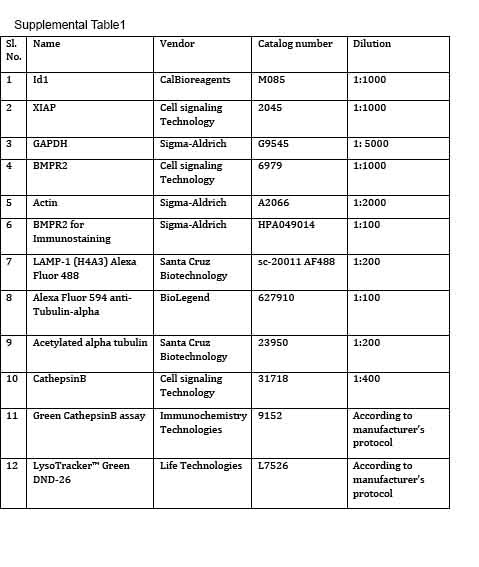

Supplement: Supplementary file 2 — Additional file 1. JL5 and Ym155 together increase cathepsin B expression in cytoplasm. [file 12964_2021_743_MOESM2_ESM.jpg]

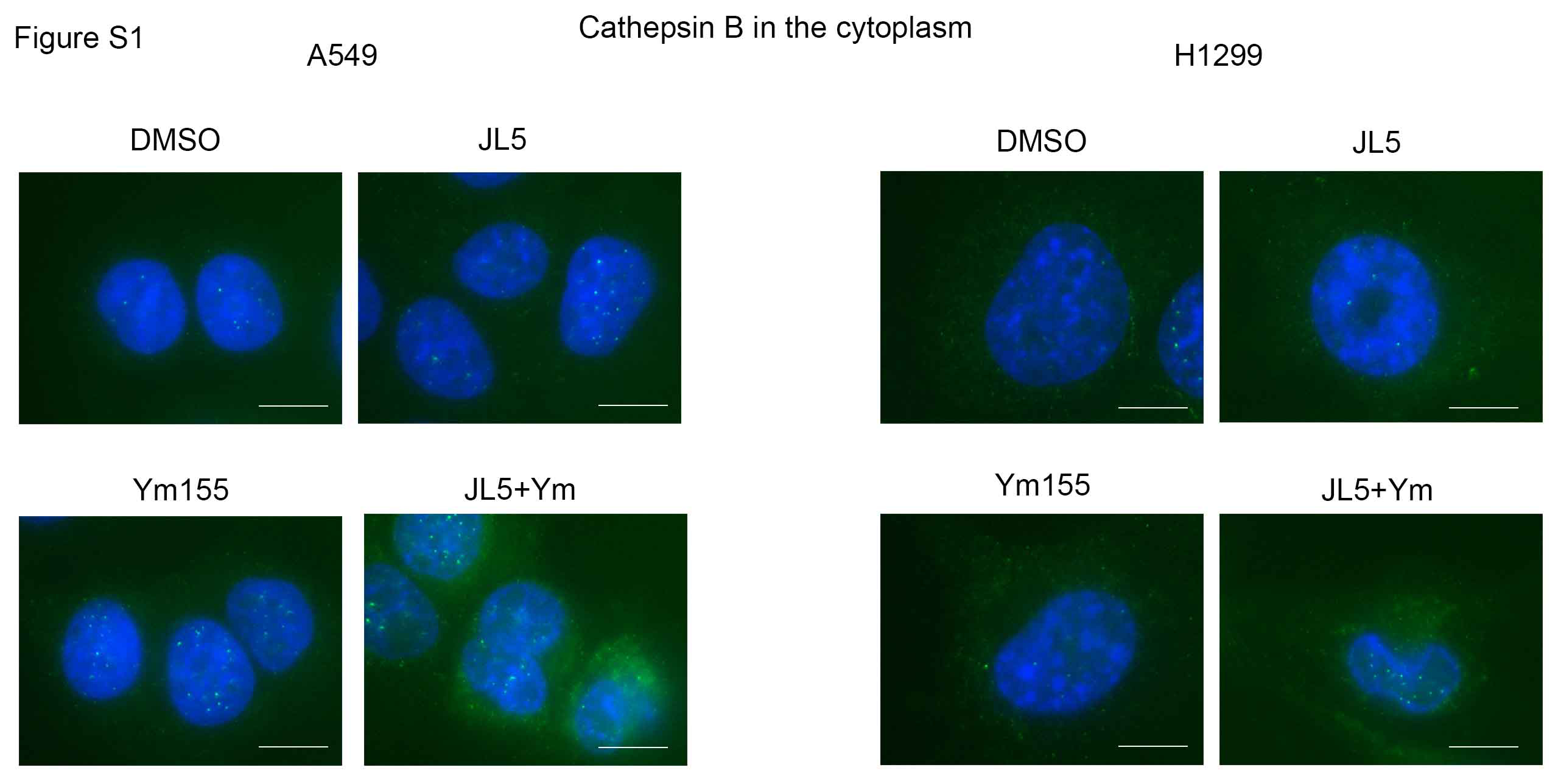

Supplement: Supplementary file 3 — Additional file 2: Fig. S1. The combination of JL5 together with Ym155 increases expression of Cathepsin B in the cytoplasm. (A-B) Immunofluorescent imaging for Cathepsin B of A549 and H1299 cells treated with JL5 2.5 uM and Ym155 20 nM alone and in combination for 24 hr. Arrows show the expression of Cathepsin B in the cytoplasm. Each scale bar represents 10 μM. [file 12964_2021_743_MOESM3_ESM.jpg]
